# Family psychoeducation to improve outcome in caregivers and patients with schizophrenia: a randomized clinical trial

**DOI:** 10.3389/fpsyt.2023.1171661

**Published:** 2023-06-23

**Authors:** Arnaud Tessier, Karine Roger, Alexandra Gregoire, Pauline Desnavailles, David Misdrahi

**Affiliations:** ^1^Department of Adult Psychiatry, Charles Perrens Hospital, Bordeaux, France; ^2^Aquitaine Institute for Cognitive and Integrative Neuroscience, Bordeaux, France; ^3^Fondation Fondamental, Créteil, France

**Keywords:** psychoeducation, caregivers, schizophrenia, relapse, burden, depression, therapeutic alliance, quality of life

## Abstract

**Introduction:**

Schizophrenia is recognized for its severe impact on both patients and caregivers. In a 12-month follow-up randomized clinical trial, we aimed to measure the efficacy of a brief family psychoeducation program in terms of reducing relapse risk and improving medication adherence in patients, as well as reducing caregiver burden, depression and increasing knowledge of the illness.

**Methods:**

A total of 25 days of patients with schizophrenia (DSM-IV-TR) and family primary caregivers were recruited in a single regional psychiatric outpatient facility located in Bordeaux. In the active group, caregivers received a psychoeducational intervention consisting of six sessions spread over 1.5 months, while the control group was placed on a waiting list. Sociodemographic, symptom severity (PANSS) and medication adherence (MARS) from patients were assessed at baseline and relapse rates was recorded during the 12 months follow-up period. Caregivers’ burden (ZBI), depression (CES-D), quality of life (S-CGQoL), knowledge of the disease (KAST) and therapeutic alliance (4PAS-C) were assessed at baseline, three and 6 months.

**Results:**

On the 25 patients included, the mean age was 33.3 years (SD = 9.7) with a mean duration of disease of 7.48 years (SD = 7.1). On the 25 caregivers included, the mean age was 50.6 years (SD = 14.0). Twenty-one were female (84.0%), 12 were married (48.0%) and 11 lived alone (44.0%). For patients, the family psychoeducation intervention significantly reduced the risk of relapse with a significant effect found at 12 months follow-up (*p* = 0.014). No change was observed on medication adherence. For caregivers, the intervention reduced the burden (*p* = 0.031), decreased the depression (*p* = 0.019), and increased the knowledge on schizophrenia (*p* = 0.024). Analyzes for repeated measures showed a statistically significant difference in therapeutic alliance (*p* = 0.035).

**Conclusion:**

As confirmed by previous studies, the brief multifamily program (consisting of six sessions over a period of 1.5 months) was found to be effective in improving outcomes for caregivers (e.g., burden, depression, knowledge) and patients (e.g., preventing relapse) in the context of routine care. Given its short duration, this program is expected to be easily implementable within the community.

**Clinical trial registration:**

https://clinicaltrials.gov/, NCT03000985.

## Introduction

Schizophrenia is a chronic and severe mental disorder which has serious consequences for both the patient and caregivers. The burden of schizophrenia on caregivers had been demonstrated (1–4) and justify that family have to be included in the care plan with adequate information and support (5). Therefore, family intervention should be developed to reduce the burden of caregivers and enhance patients’ prognosis. For patients, family psychoeducation has been effective in improving outcomes in schizophrenia with a better level of global functioning, medication adherence, and a reduction in the use of healthcare resources and the frequency of relapse (6–8). A recent systematic review including 11 studies demonstrates consistent improvement in many outcome measures of patients, such as relapse rates and medication adherence, but heterogeneity in symptoms reliefs (9). Its effectiveness has also been demonstrated for individuals at clinical high risk for psychosis although rigorous further studies are required (10). Through psychoeducation, a better understanding of the illness was associated with a better insight and medication adherence (11). For caregivers, increased knowledge of the disease reduces aspects related to stigma, stress and burden which contributes to a supportive social environment to increase the patient’s awareness of the disease and adapted care (12, 13).

It has been demonstrated that family psychoeducation is effective and is considered part of the guideline recommendations in the treatment of schizophrenia (5, 14, 15). A Cochrane review confirmed a 20% reduction in relapse rates compared with usual care (7). Caregivers’ outcomes from family psychoeducation are less commonly studied. The only meta-analysis of family outcomes found considerable positive effects on relatives’ burden and psychological distress, the relationship between relatives and the patient, and family functioning (16). A review of family psychoeducation programs suggested that it was more likely to be effective in families if knowledge of the disease and other outcomes such as burden, family functioning, emotional response etc. were systematically assessed to reflect the specific goals of the intervention (8). Despite recommendations and significant results, family psychoeducation is not a widely accessible option in mental health services, often due to limitations including a lack of interest from families, limited availability of care staff, or a shortage of trained professionals in these programs (5, 17, 18). A review of more than 30 randomized clinical trials (RCTs) pointed out that application of family psychoeducation in routine settings remains limited, reflecting attitudinal, knowledge, practice and systemic barriers to implementation (19).

The objective of this study was to compare the efficacy of a new brief family psychoeducation program (consisting of 6 sessions over 1.5 months) to treatment as usual (TAU) in a single center randomized clinical trial. Our aim was to measure the impact of the intervention on both caregivers and patients over a 12-month follow-up period, in comparison to usual care. We hypothesized that the psychoeducation program would reduce the risk of relapse and improve medication adherence in patients with schizophrenia, while also enhancing the quality of life, reducing caregiver burden, improving the therapeutic alliance, increasing knowledge of the illness, and reducing depression in caregivers.

## Methods

### Study site and participants

This randomized single-blind controlled trial adopted a two arms parallel groups design. The controlled trial was conducted at a single regional psychiatric outpatient facility located in Bordeaux, France, registered in ClinicalTrials.gov (Ref.: NCT03000985). A total of 25 dyads of patients and family primary caregivers were recruited between December 2014 and December 2019. The inclusion criteria for patients were (i) a diagnosis of schizophrenia or schizoaffective disorder according to DSM-IV-TR criteria (20), (ii) age of at least 18 years, and (iii) being in a stable phase and receiving outpatients’ routine care. Exclusion criteria consisted of a history of traumatic head injury, any current or past major medical or neurological illness, and mental retardation. The inclusion criteria for caregivers were males or females aged 18 years or older who were currently caring for a relative diagnosed with schizophrenia or schizoaffective disorder and receiving appropriate outpatient clinical care. Caregivers who had previously received a standardized psychoeducational intervention or had intellectual disability, dementia or any other psychiatric condition were excluded from the study. Participants were assigned to one of the two study groups through a computer-generated random process. On the 25 caregivers 11 were the mother, 7 the father, 2 the sister, 1 the brother, 1 spouse/husband, 1 the aunt and finally 2 were the child of patient. In the active group, caregivers received a psychoeducational intervention consisting of six sessions spread over 1.5 months, while the control group, which received treatment as usual (TAU), was placed on a waiting list without any additional strategies. All participants including patients and caregivers signed an informed consent form prior to randomization and trial inclusion. The study protocol involving human participant was reviewed and approved by the local human subject research ethics committee.

### Intervention

The *Schiz’Aides* program is a multifamily psychoeducational program, which was built for caregivers of patients with schizophrenia, consisting of six sessions over a 1.5 months period. Each session lasted an average of 1 h and 30 min and was delivered in a group format led by a nurse and a psychologist who were trained to provide psychoeducation. One specific session of the program included the participation of a psychiatrist and a social worker. The program follows international guidelines concerning these interventions (5). The first session focuses on the presentation of each family and the experience of their relative’s illness. It also provides an opportunity to gather families’ expectations and to present the program’s themes. The second session is dedicated in understanding the disease. The objective is to give caregivers the criteria to identify symptoms, as well as etiological factors (multifactorial hypothesis). The third session focuses on drug treatments (role, forms, efficacy, side effects), non-drug treatments (mainly psychotherapies such as cognitive remediation) and finally forms/modes of hospitalization. The fourth session allows the caregiver to better manage their patients’ crisis states. The objective is to identify the warning signs and the adaptive reaction to adopt. The sharing of experiences between the different families is strongly encouraged. The fifth session focuses on the patient’s daily life, i.e., the psychosocial and cognitive consequences, as well as negative symptoms hindering a good rehabilitation. Solutions for care, networks and associations are presented. The sixth session focuses on the caregivers’ experiences: the weight of the disease, verbalization of feelings. Afterward, a review of the program is made and answers to the last questions are given. A booster session is conducted six months after the initial phase. This session allows to improve the program’s efficacy and to monitor the implementation of daily effective strategies. The *Schiz’Aides* program has a complete manual with a session guide, which can be provided upon request.

### Data collection

#### Patient’s assessment

Patients were assessed once at inclusion. Sociodemographic information (gender, age, marital status, level of education, living situation) and illness history (duration of illness, lifetime suicide attempt, history of treatments, BMI) were collected. Symptom severity was assessed using the Positive And Negative Syndrome Scale (PANSS) (21) and medication adherence was evaluated with the Medication Adherence Rating Scale (MARS) (Kuder–Richardson-20: 0.60) (22). With the agreement of the authors (23), our team translated the scale into French, then validated it by reverse translation into English with the author. The scale was then used and validated in a large national cohort of 319 patients suffering from schizophrenia (24, 25). Relapses, defined as new psychiatric hospitalization, were recorded by reviewing the computerized medical records at 3, 6, and 12 months from baseline.

#### Caregivers’ assessment

Caregivers were assessed at baseline, and at 3 and 6 months after the completion of the psychoeducational program. Self-administered questionnaires were used to assess the different dimensions: the knowledge of the disease using the Knowledge About Schizophrenia Test (KAST). The KAST is an 18-item multiple-choice questionnaire (e.g., “Medicines that are used for hearing voices are called …”; “A person strongly believes that the FBI has put a computer chip in his/her body. This symptom is called a …”) with five response options and the score ranges from 0 to a maximum possible score of 21 (indicating good knowledge) (Kuder–Richardson-20: 0.82) (26); the Quality of Life with Schizophrenia-Caregiver Quality of Life questionnaire (S-CGQoL) contains 25 items scored with a six-point Likert scale and describing seven dimensions, with 100 indicating the best possible level of QoL and 0 the worst (27); the burden of disease was assessed with the Zarit Burden Interview (ZBI). The ZBI includes 22 statements (e.g., “Do you feel that because of the time you spend with your relative that you do not have enough time for yourself?”; “Do you feel your health has suffered because of your involvement with your relative?”) recorded in a 0–4 Likert scale (total score range 0 to 88), that rates the subjective component of burden (Cronbach’s alpha: 0.92) (28); the Center for Epidemiologic Studies – Depression Scale (CES-D) is a 20-item questionnaire that measures depressive symptoms and related behaviors experienced over the past week, with each item rated on a 0–3 Likert scale. Possible scores range from 0 to 60, with higher scores indicating more severe depressive symptoms. A cutoff score of 16 or greater is indicative of individuals at risk for clinical depression (Cronbach’s alpha: 0.90) (29); the 4-Point ordinal Alliance Scale – Caregiver (4PAS-C) is an 11-item questionnaire (e.g., “I believe my doctor is helping us”; “I have a better understanding of the symptoms of my relative’s illness.”) scored using a Likert-type format. Responses range from 1 (“strongly disagree”) to 4 (“strongly agree”) and scores range from 11 to 44, with higher scores indicating of a more positive alliance (Cronbach’s alpha: 0.91). The questionnaire yields two subscores (Empathy and Psychoeducation) and a Visual Analog Scale score (0 to 100) (30); the Compliance Rating Scale (CRS), a seven-point rating scale, score ranging from 1 (complete refusal) to 7 (active participation) (31).

### Statistical analysis

Analyzes were conducted based on the “intention-to-treat” principle; patients and caregivers were analyzed according to the randomization group they were allocated to and regardless of the intervention they followed. Baseline characteristics of patients and caregivers were compared between intervention and control groups using appropriate tests. They were no dropout at follow-up visits. When normality assumption was not rejected, an independent t-test was used for continuous variables. Otherwise, the Mann–Whitney U test was performed. Categorical data were compared using a chi-squared test or a Fisher’s exact test if necessary. Longitudinal repeated measures (baseline, 3 and 6 months) of caregivers’ scales were analyzed in each randomized group using whether repeated measures ANOVA (for normal distributed data) or Friedman test (for non-normal distributed data), followed by a post-hoc analysis using the Wilcoxon signed-rank test with Benjamini-Hochberg correction.

All *p* values were two-sided, and the level of statistical significance was set to 5%. Statistical analyzes were performed with IBM SPSS statistics, version 26.0. Complete test statistics are displayed in the respective tables and Supplementary data.

## Results

### Sample characteristics at baseline

On the 25 patients included, the mean age was 33.3 years (SD = 9.7). They were 6 females (24.0%) with a mean duration of disease of 7.48 years (SD = 7.1). The majority were single (*n* = 24, 96%), lived alone (*n* = 15, 60%) and were unemployed (n = 22, 88%). Regarding the clinical variables, patients had a mean duration of illness of 7.5 years (SD = 7.1), a mean number of antipsychotics of 1.20 (SD = 0.5) and a mean antipsychotic dose of 4.55 mg (SD = 4.2) chlorpromazine equivalent. All patients were treated with a second-generation antipsychotic. The mean total PANSS score was 70.76 (SD = 11.8) and the mean score on the MARS scale was 6.52 (SD = 1.9) (moderate compliance). At baseline the two study groups were significant different on PANSS scores, antipsychotic dose, and BMI. The sociodemographic and clinical characteristics of the sample are presented in Table 1.

**Table 1:** Demographic and clinical characteristics of patients and caregivers included in the study.

	Patients			Caregivers		
	Fisher’s exact test			Fisher’s exact test		
	Active (PsyEduc) (*N* = 12)	Control (TAU) (*N* = 13)		Active (PsyEduc) (*N* = 12)	Control (TAU) (*N* = 13)	
VARIABLES	*n* (%)	*n* (%)	*p*	*n* (%)	*n* (%)	*p*
Gender, male	8 (66.7)	11 (84.6)	0.561	3 (25.0)	1 (7.7)	0.603
Marital status, married	0 (0.0)	1 (7.7)	1.000	8 (66.7)	4 (30.8)	0.567
Housing, alone (vs. accompanied)	8 (66.7)	7 (53.8)	0.806	8 (66.7)	3 (23.1)	0.284
Level of education, high school or lower	9 (75.0)	7 (53.8)	0.494	1 (8.3)	2 (15.4)	0.838
Employment status, work (vs. unemployed)	1 (8.3)	2 (15.4)	1.000			
Second generation of antipsychotics (vs. first)	11 (91.7)	10 (76.9)	0.593			
	Mann–Whitney U-test for independent samples			Mann–Whitney U-test for independent samples		
	Mean (SD)	Mean (SD)	*p*	Mean (SD)	Mean (SD)	*p*
Age (years)	36.17 (11.3)	30.62 (7.7)	0.161	45.92 (15.7)	56.89 (8.5)	0.055
BMI (kg/m^2^)	**27.06 (5.0)**	**24.06 (3.5)**	**0.034** *			
Number of children	0.17 (0.4)	0.00 (0.0)	0.148			
Duration of illness (years)	7.83 (6.2)	7.15 (8.0)	0.298			
Number of suicide attempts (lifetime)	0.08 (0.3)	0.15 (0.4)	0.629			
Average number of antipsychotics	1.25 (0.6)	1.15 (0.4)	0.898			
Antipsychotic dose (1 mg chlorpromazine equivalent)	**3.54 (4.0)**	**5.48 (4.3)**	**0.043** *			
PANSS	**64.58 (10.2)**	**76.46 (10.5)**	**0.009** *			
Positive symptoms	**13.50 (3.6)**	**18.77 (4.3)**	**0.003** *			
Negative symptoms	17.50 (4.9)	19.31 (6.7)	0.453			
General psychopathology	**33.58 (4.5)**	**38.38 (4.0)**	**0.010** *			
MARS	6.78 (1.7)	6.33 (2.0)	0.601			
Medication adherence behavior	2.67 (0.9)	2.67 (1.1)	0.969			
Attitude to taking medication	3.11 (0.6)	2.83 (1.3)	0.970			
Negative side-effects	1.00 (1.0)	0.83 (0.9)	0.726			

Mean (SD): mean +/− standard deviation.

BMI, Body Mass Index; PANSS, Positive and Negative Syndrome Scale; MARS, Medication Adherence Rating Scale.

* Significant difference with *p* < 0.05 are in bold text.

On the 25 caregivers included, the mean age was 50.6 years (SD = 14.0). Twenty-one were female (84.0%), 12 were married (48.0%) and 11 lived alone (44.0%). Twenty-two had a level of education higher than the high school (88%). The mean quality of life was 70.80 (SD = 16.5). Burden was scored as medium with a mean of 37.75 (SD = 7.9). Knowledge of the disease was rated as “moderate” with a mean score of 13.1 (SD = 2.3) and therapeutic alliance was rated as “good” with a mean sore of 30.94 (SD = 7.4). Depression was rated as “moderate” with a mean score on CES-D of 20.37 (SD = 11.4). No significant difference was found between groups for sociodemographic and psychometrics scores of caregivers at baseline. A positive significant association was found, between the burden (ZBI) and the level of depression (CES-D) [*β* = 0.78, CI = (0.06, 1.50), *p* = 0.036].

### Patient’s outcome

A lower rate of relapse was observed at 3, 6 and 12 months for patients whose caregivers participated in the intervention group. The difference was significant at 12 months (*p* = 0.014). Medication adherence assessed by CRS estimated by caregivers was not modified by the intervention (see details in Table 2).

**Table 2:** Comparison of patients’ outcomes (cumulative relapse (re-hospitalization) rates and perceived medication adherence) following time.

	M3			M6			M12		
	Active (PsyEduc) (*N* = 12)	Control (TAU) (*N* = 13)		Active (PsyEduc) (*N* = 12)	Control (TAU) (*N* = 13)		Active (PsyEduc) (*N* = 12)	Control (TAU) (*N* = 13)	
	*n* (%)	*n* (%)	*p*	*n* (%)	*n* (%)	*p*	*n* (%)	*n* (%)	*p*
Cumulative relapses	0 (0.0)	3 (37.5)	0.058	0 (0.0)	3 (37,5)	0.082	0 (0.0)	4 (50.0)	**0.014** *
	Mean (SD)	Mean (SD)	*p*	Mean (SD)	Mean (SD)	p	Mean (SD)	Mean (SD)	*p*
CRS	6.22 (1.4)	5.88 (1.6)	0.778	5.82 (1.7)	6.13 (1.5)	0.747	5.80 (2.2)	6.75 (0.5)	0.661

SD, Standard Deviation; CRS, Compliance Rating Scale.

Percentages are adjusted for lost and missing data.

* Significant difference with *p* < 0.05 are in bold text.

### Caregiver’s outcome

The results indicated a significant difference in the total score of the KAST at 1.5 months (*p* = 0.024). Caregivers who received psychoeducation had higher scores (mean: 15.45; SD: 1.4) than caregivers in the control group (mean: 12.38; SD: 3.4). This difference was not observed at 6 months (*p* = 0.098). Two significant results were found at 6 months: first, the ZBI score (burden) (*p* = 0.031), showed a significant reduction for caregivers in the active group (mean: 20.17; SD: 8.0) compared to the control group (mean: 35.60; SD: 12.1); secondly, the CES-D score (depression) showed a significant reduction (*p* = 0.019), for caregivers in the active group (mean: 6.20; SD: 4.9) compared to the control group (mean: 14.20; SD: 3.6). No significant differences were found for quality of life and therapeutic alliance, regardless of the visit. Additional details are provided in Table 3.

**Table 3:** Comparisons of caregiver’s outcomes (scores on psychometric scales) at each visit.

	Mann–Whitney U-test for independent samples								
	M0			M3			M6		
	Active (PsyEduc)	Control (TAU)		Active (PsyEduc)	Control (TAU)		Active (PsyEduc)	Control (TAU)	
	(*N* = 12)	(*N* = 13)		(*N* = 12)	(*N* = 13)		(*N* = 12)	(*N* = 13)	
VARIABLES	Mean (SD)	Mean (SD)	*p*	Mean (SD)	Mean (SD)	*p*	Mean (SD)	Mean (SD)	*p*
S-CGQoL	73.17 (20.5)	67.25 (7.4)	0.376	79.64 (20.3)	74.62 (6.6)	0.458	87.83 (14.8)	70.60 (14.0)	0.080
Psychological and physical well-being	15.00 (4.6)	12.00 (3.3)	0.128	15.55 (4.3)	14.00 (4.4)	0.452	19.17 (2.6)	13.60 (6.1)	0.073
Psychological burden and daily life	21.25 (7.5)	20.38 (6.8)	0.794	23.45 (6.2)	23.25 (6.6)	0.946	24.83 (3.5)	20.00 (8.2)	0.221
Relationships with spouse	8.25 (5.6)	5.75 (5.8)	0.415	8.18 (5.3)	6.00 (5.6)	0.405	10.33 (4.1)	6.00 (5.0)	0.149
Relationships with psychiatric team	8.00 (4.9)	9.88 (3.6)	0.367	10.45 (3.6)	9.00 (3.9)	0.415	11.00 (4.3)	10.80 (4.0)	0.939
Relationships with family	6.50 (2.6)	4.63 (3.8)	0.204	6.36 (2.8)	6.50 (2.8)	1.000	7.83 (2.0)	5.60 (3.2)	0.194
Relationships with friends	5.67 (3.3)	5.25 (3.2)	0.783	7.44 (2.4)	6.25 (2.9)	0.375	6.67 (2.1)	5.40 (3.4)	0.468
Material burden	8.50 (6.3)	9.38 (2.3)	0.666	8.18 (6.1)	9.63 (2.9)	0.867	8.00 (6.8)	9.20 (1.8)	0.692
ZBI	37.67 (17.3)	37.88 (19.9)	0.980	30.45 (16.9)	35.38 (21.4)	0.582	**20.17 (8.0)**	**35.60 (12.1)**	**0.031** *
KAST	13.67 (1.6)	12.25 (3.1)	0.192	**15.45 (1.4)**	**12.38 (3.4)**	**0.024** *	15.33 (1.9)	12.80 (2.7)	0.098
4PAS-C	30.30 (7.8)	31.75 (7.1)	0.691	36.67 (7.8)	35.50 (6.8)	0.749	36.40 (10.7)	32.50 (9.9)	0.593
Visual analogic scale	50.50 (34.3)	49.38 (30.5)	0.943	71.11 (25.0)	67.50 (26.3)	0.776	71.00 (34.0)	49.50 (46.2)	0.321
Empathy	14.20 (3.4)	15.50 (3.4)	0.435	17.11 (3.3)	16.63 (3.1)	0.626	17.00 (5.2)	15.00 (4.2)	0.521
Psychoeducation	16.10 (4.6)	16.25 (3.9)	0.942	19.56 (4.5)	18.88 (3.9)	0.745	19.40 (5.7)	17.50 (5.9)	0.641
CES-D	20.27 (13.3)	20.50 (8.9)	0.967	14.91 (10.0)	23.54 (11.3)	0.118	**6.20 (4.9)**	**14.20 (3.6)**	**0.019** *

SD, Standard Deviation; S-CGQoL, Schizophrenia Caregiver Quality of Life questionnaire; ZBI, Zarit Burden Interview; KAST, Knowledge About Schizophrenia Test; 4PAS-C, 4-Point ordinal Alliance Scale–Caregiver; CES-D, Center for Epidemiologic Studies–Depression scale.

* Significant difference with *p* < 0.05 are in bold text.

Analyzes for repeated measures showed a statistically significant difference in therapeutic alliance (4PAS-C total score) (*p* = 0.035) and two sub-scores: Visual Analogic Scale (*p* = 0.015), and Psychoeducation (*p* = 0.036). There was also a significant improvement in the burden, as evaluated by ZBI (*p* = 0.040; see Table 4 for details). Post hoc analysis did not yield any significant results, despite an overall perceived improvement of therapeutic alliance between caregiver and the healthcare team and a reduction in the burden (Supplementary data).

**Table 4:** Evolutions in psychometric scales’ scores by group: Friedman’s ANOVA for repeated measures.

		S-CGSQoL								ZARIT	KAST	4PAS-C				CES-D	CRS
	Friedman’s ANOVA	Total	PsPhW	PsBDL	RS	RPT	RFa	RFr	MB	Total	Total	Total	VAS	Em	PE	Total	Total
Active (PsyEduc)	Khi-square	0.785	1.634	0.290	0.231	1.433	0.556	0.900	0.545	**4.507**	3.756	**5.218**	**7.354**	2.000	**5.167**	1.852	0.545
	*p*	0.482	0.243	0.754	0.798	0.284	0.590	0.638	0.761	**0.040** *	0.061	**0.035** *	**0.015** *	0.368	**0.036** *	0.236	0.761
Control (TAU)	Khi-square	1.071	0.161	0.498	0.054	0.615	3.797	2.308	1.000	0.460	0.762	0.743	0.594	5.167	1.588	1.476	2.000
	*p*	0.387	0.854	0.626	0.948	0.564	0.069	0.200	0.410	0.647	0.498	0.515	0.497	0.755	0.297	0.291	0.368

S-CGQoL–PsPhW, Psychological and Physical Well-being; PsBDL, Psychological Burden and Daily Life; RS, Relationships with Spouse; RPT, Relationships with Psychiatric Team; RFa: Relationships with Family; RFr: Relationships with Friends; MB: Material Burden; Index, global S-CGQoL score. S-CGQoL scores, ranging.

from 0 to 100 (with 100 indicating best quality of life).

4-PAS-C–VAS, Visual Analogic Scale; Em, Empathy; PE, Psychoeducation.

* Significant difference with *p* < 0.05 are in bold text.

## Discussion

The efficacy of a family psychoeducational program was assessed through a randomized clinical trial (RCT) on a community dwelling patient with schizophrenia and their caregivers. Principal findings should be summarized as follows: (i) For patients, the family psychoeducation intervention reduced the risk of relapse with a significant effect found at 12 months follow-up. However, no change was observed on medication adherence; (ii) For caregivers, the intervention reduced the burden, decreased the depression, increased the knowledge on schizophrenia, and strengthened the therapeutic alliance.

The efficacy of family psychoeducation in patient with schizophrenia has been well established in previous studies (9, 19). A review of the literature showed that family intervention can improve relapse and hospital admission rates in early psychosis (32). Additionally, studies conducted in chronic schizophrenia found that family psychoeducation can reduce the risk of relapse (25, 26, 33, 34). These interventions have also been shown to be cost saving and are included in international treatment guidelines (14, 15). These findings highlight the importance of incorporating family psychoeducation as a part of the comprehensive treatment plan for patients with schizophrenia.

Despite expectations, we did not show any impact of the family psychoeducation on medication adherence. This result is consistent with a previous study where carers’ knowledge about schizophrenia appeared to be not related to compliance (35). Medication adherence which is recognized as complex and multi-determined phenomenon cannot be resolved by a single, non-specific intervention. Moreover, the caregiver’s judgment of their relative’s medication intake may be influenced by factors such as the amount of time spent together and the patient’s regimen (oral or injectable antipsychotic). This presents a limitation for the interpretation of scores on the CRS and highlights the need for a combination of objective measures (e.g., pill counts, serum levels) and validated self-report scales to accurately assess medication adherence (25, 36). Nevertheless, the level of caregiver burden may also have an impact on medication adherence, as demonstrated by recent studies, which underscore the importance of supportive programs for caregivers (9, 37).

In caregivers we found at baseline a significant association between the level of depression and the burden estimated with the Zarit Burden Interview. This association is confirmed by the study from Mittendorfer-Rutz (4) in a nationwide comparative study of parents of offspring with schizophrenia, rheumatoid arthritis, multiple sclerosis, epilepsy, and healthy controls. The results of this study showed that the parents of a patient with schizophrenia were at a higher risk for burden and had a 2.7 times higher risk of needing specialized psychiatric health care. We found a significant effect in caregiver’s burden at 6 months follow-up, suggesting a temporal learning effect with a gradual reduction in burden over time attributed to the family psychoeducation program. The impact of schizophrenia on caregivers can result in a significant burden including emotional, psychological, physical and financial strain associated with feelings of shame, embarrassment, guilt and blame (16, 37). Reducing caregiver burden is crucial for patient management, and for the caregiver himself, as it can help to reduce depression, burden-related care and associated costs (38).

At baseline, the level of depression is evaluated as “moderate” in both groups, with a score above the threshold fixed at 16 on CES-D scale. Previous studies had shown that caregivers of patients with schizophrenia are at higher risk for developing depression (4, 38). In a large survey, Gupta et al. found a 10% increased of depression in caregivers of patients with schizophrenia compared to non-caregivers and caregivers of adults with other conditions (38). At 6 months, in comparison with TAU, the intervention was found to significantly reduce depression. Depressive symptoms can have a negative impact on family interactions and lead to maladaptive behaviors toward the patient. Our findings is consistent with a previous RCT showed the usefulness of a family intervention in reducing caregiver’s depressive symptoms as measured by the CES-D, and a moderate effect in reducing the subjective burden as assessed by the ZBI over an 8 months follow-up period (2).

The purpose of family psychoeducation is to increase caregiver’s knowledge and understanding of illness and treatment. A significant improvement in the knowledge of the disease (KAST) was found in caregivers in the intervention group. This demonstrates the relevance of the information delivered during psychoeducation and its directly measurable effect (33). However, at 6 months, the difference between the two groups was no longer significant. There was a spontaneous improvement in the score in the control group, which may be related to the caregivers’ self-training by different resources (books, internet, meeting with the treating psychiatrist...). Knowledge of the disease have been found to be associated with a better medication adherence in patients (11). The Cochrane review highlights the benefits of patient psychoeducation in reducing relapse and readmission rates, promoting medication adherence, and shortening hospital stays. These findings suggest that psychoeducation not only has a positive impact on patients, but also on their family caregivers, making it a clinically effective and cost-beneficial intervention (39). The central role of family support in care was recently coroborated in an Italian multicenter study of 136 caregivers, where caregivers’ personal growth was associated with good family functioning and adequate professional support (40). Another study focused on the functioning pointed out that interpersonal relationships and work skills are the impaired functional areas in both patients and caregivers (41).

Therapeutic alliance has been found to be enhanced by the family psychoeducation intervention. To our best knowledge, the therapeutic alliance between caregivers and healthcare staff has never been investigated in cohorts of patients with schizophrenia. Interactions with the health care staff during the family psychoeducation group strengthened the relationship and understanding with the caregiver. This was explored in other pathologies such as cancer (42).

In contrast to the study by Savanaud et al., we did not find any impact of the intervention on quality of life (38, 43). Quality of life may be influenced by the heterogeneous relational degree of the caregiver (parents, partner, child…). Indeed, it has been shown that parents of a patient have a lower quality of life than the patient’s siblings as demonstrated in a recent Croatian study (44). Although 72% of the included caregivers in our sample were the ill loved one, no significant results emerged regarding their quality of life. Most studies with similar design have focused on the patient’s quality of life rather than the caregiver’s (45, 46). We found one recent Indian RCT, that showed a significant improvement in overall quality of life scores in the experimental group caregivers compared to control group at the end of the program, after 6 months (47).

Thus, our study provides evidence of the efficacy of a short multifamily program (six sessions over 1.5 months) for caregivers (depression, knowledge) and patients (preventing relapse) in the context of routine care. This program can be repeated multiple times during the year. Previous RCTs and Evidence-Based Medicine (EBM) have considered the same primary evidence (9); however, the implementation of this evidence in routine care is limited (19). Moreover, To the best of our knowledge, and within the context of French mental health, psychoeducational programs for patients have been more extensively developed than those for caregivers. The brevity of this program should facilitate its implementation in the community.

### Limitations

Although our study has several strengths, there are some limitations that must be acknowledged. Our limited sample size could limit the reliability of our results. Additionally, the single-center design of our study may introduce a selection bias, which may affect the representativeness of our sample. To fully understand the effects of the intervention and to confirm our encouraging results, a larger, multi-center study should be done. However, in the context of the French mental health psychiatric service, the regional psychiatric outpatient facility should be considering as a good representation of the population of a community dwelling patient suffering of schizophrenia. Although patients were considered to be in a stable phase with only minor modifications to their prescribed medication during follow-up, this issue was not controlled and should be considered as a limitation in assessing the effectiveness of the psychoeducational program in preventing relapse.

In conclusion, as confirmed by previous studies, the brief multifamily program (consisting of six sessions over a period of 1.5 months) was found to be effective in improving outcomes for caregivers (e.g., burden, depression, knowledge) and patients (e.g., preventing relapse) in the context of routine care. Given its short duration, this program is expected to be easily implementable within the community.

## Data availability statement

The raw data supporting the conclusions of this article will be made available by the authors, without undue reservation.

## Ethics statement

The studies involving human participants were reviewed and approved by Committee for the Protection of Persons–South West and Overseas 3 (CPP-SOOM3) Study folder: 2014/58. Department of Clinical Pharmacology, Bordeaux Hospital, Place Amélie Raba Léon 33076 BORDEAUX Cedex FRANCE Tel: +33 (0)5 57 81 76 07 Email: cpp.soom3@u-bordeaux.fr Web: http://www.cpp-soom3.u-bordeaux2.fr/. The patients/participants provided their written informed consent to participate in this study.

## Author contributions

KR and DM were involved in generating hypotheses, the management of the study and the development of the psychoeducational program. KR, AG, and DM were involved in the conduction of the psychoeducational program (Schiz’Aides). AT and PD conducted statistical analyzes. AT, PD, and DM wrote the first complete manuscript. All authors were involved in the patients’ recruitment, the clinical evaluation, acquisition of the clinical data, modified the manuscript and approved the final version.

## Funding

This work was supported by a grant from the Nurses and Paramedics Research Hospital Program from the French Ministry of Health (PHRIP “2013–0017–PsyEduc”). The funding source had no role in the conduct or publication of the study.

## Conflict of interest

The authors declare that the research was conducted in the absence of any commercial or financial relationships that could be construed as a potential conflict of interest.

## Publisher’s note

All claims expressed in this article are solely those of the authors and do not necessarily represent those of their affiliated organizations, or those of the publisher, the editors and the reviewers. Any product that may be evaluated in this article, or claim that may be made by its manufacturer, is not guaranteed or endorsed by the publisher.
