# Supplementary material for: Family psychoeducation to improve outcome in caregivers and patients with schizophrenia: a randomized clinical trial
Source: Front Psychiatry. 2023 Jun 23;14:1171661. doi: 10.3389/fpsyt.2023.1171661 (PMC10326382; doi:10.3389/fpsyt.2023.1171661)
Supplement: Supplementary file 1 [file Table_1.docx]

Supplementary Material

Family psychoeducation to improve outcome in caregivers and patients with schizophrenia: a randomized clinical trial.

Arnaud TESSIER^*^, Karine ROGER, Alexandra GREGOIRE, Pauline DESNAVAILLES, David MISDRAHI

*** Correspondence:**Arnaud TESSIER
atessier@ch-perrens.fr

# Supplementary Figures and Tables

**Table Supp. 1**

Post-hoc tests for comparison between visits on variables significant to Friedman's ANOVA (with a Benjamini-Hochberg correction).

|  |  | | **M0 *vs*. M3** | **M3 *vs*. M6** | **M0 *vs*. M6** |
| --- | --- | --- | --- | --- | --- |
| **Active**  **(PsyEduc)** | ZBI  total | Wilcoxon Z | 2.214 | 3.220 | 2.297 |
|  |  | p | 0.070 | 0.070 | 0.070 |
|  | 4PAS-C  total | Wilcoxon Z | -2,556 | 1.089 | -2.304 |
|  |  | p | 0.113 | 0.338 | 0.124 |
|  | 4PAS-C  Visual Analogic Scale | Wilcoxon Z | -3.035 | 0.272 | -2.638 |
|  |  | p | 0.057 | 0.799 | 0.087 |
|  | 4PAS-C  Psychoeducation | Wilcoxon Z | -2.308 | 0.885 | -2.631 |
|  |  | p | 0.087 | 0.426 | 0.087 |

ZBI: Zarit Burden Interview; 4PAS-C: 4-Point ordinal Alliance Scale – Caregiver.

* : significant difference with p < 0.05 are in bold text.
